# Spectrum of Fontan-associated liver disease assessed by MRI and US in young adolescents

**DOI:** 10.1007/s00261-021-02994-0

**Published:** 2021-03-10

**Authors:** Karl Julius Thrane, Lil Sofie Ording Müller, Kathrine Rydén Suther, Kristian Stien Thomassen, Henrik Holmström, Erik Thaulow, Runar Almaas, Thomas Möller, Charlotte de Lange

**Affiliations:** 1 Div of Radiology and Nuclear Medicine, Section of Paediatric Radiology, Oslo University Hospital, Oslo, Norway; 2 Dept of Paediatric Cardiology, Oslo University Hospital, Oslo, Norway; 3 Dept of Paediatric Research and Div of Paediatric and Adolescent Medicine, Oslo University Hospital, Oslo, Norway; 4 Institute of Clinical Medicine, University of Oslo, Oslo, Norway; 5 Dept of Radiology and Clinical Physiology Queen Silvia Childrens’ Hospital, Sahlgrenska University Hospital, Göteborg, Sweden

**Keywords:** Diffusion-weighted imaging, Fontan procedure, Liver cirrhosis, Magnetic resonance imaging, Univentricular heart

## Abstract

**Purpose:**

Patients with Fontan circulation are at risk of developing hepatic fibrosis/cirrhosis. The mechanisms and disease development are unclear and early secondary liver cancer is a concern. This study will describe hepatic imaging findings in a national cohort of adolescents with Fontan circulation.

**Methods:**

The patients prospectively underwent abdominal contrast enhanced magnetic resonance imaging (MRI) including diffusion-weighted imaging. Images were assessed for criteria of fibrosis*/*cirrhosis including characterization of hepatic nodules. These nodules were in addition, assessed by ultrasonography (US). Nodules ≥ 1 cm were investigated and monitored to evaluate malignant transformation. Clinical and hepatic serological data were recorded.

**Results:**

Forty-six patients, median age of 16.5 years (15.4–17.9 years) were enrolled. All patients underwent US examination and MRI was performed in 35/46 patients. On MRI, 60% had hepatomegaly and 37% had signs of fibrosis/cirrhosis. Seven patients had together 13 nodules ≥ 1 cm in diameter. Only 4/13 (17%) where seen on US. Nodules had variable MRI signal characteristics including hepatobiliary contrast enhancement and two nodules revealed portal venous phase ‘wash-out’ on the first examination. No further imaging signs of malignancy were revealed during the follow-up period of median 24.4 (7–42) months.

**Conclusion:**

The majority of adolescents with Fontan circulation had imaging findings of fibrosis/cirrhosis of varying severity. US had low detection rate of hepatic nodules compared to MRI. The imaging work-up before transition to adult cardiology care did not reveal findings suggestive of malignancy. However, the high prevalence of Fontan-associated liver disease calls for surveillance strategies even in childhood.

**Supplementary Information:**

The online version of this article (10.1007/s00261-021-02994-0) contains supplementary material, which is available to authorized users.

## Introduction

Children born with univentricular heart defects are surgically palliated with the Fontan procedure, where in the United States an average of 1062 operations are performed per year [1]. Worldwide the estimation is that 70.000 patients are living with Fontan circulation. Refinements of the surgical technique and peri*/*postoperative care over the last decades have largely improved the prospects of survival into adulthood [2]. However, long-term complications of this palliative circulation are now recognized, affecting not only the heart itself but several end organs. The negative impact on the liver is of special and growing concern [3, 4].

In the Fontan circulation, one functional cardiac chamber pumps blood to the systemic side, while the systemic venous return happens passively via the caval veins, which are connected to the pulmonary arteries. This continuous non-pulsatile flow with slightly elevated central venous pressure results in a chronic hepatic congestion, with development of fibrosis*/*cirrhosis over time. The low cardiac output, and lymphatic overflow and obstruction which is caused by the increased central venous pressure hampering drainage from the thoracic duct, further inflicts hepatic hypoxic injury [5]. This multifactorial etiology leads to the hepatic condition, known as Fontan-associated liver disease (FALD) [6, 7]. As in adult chronic liver disease, there is a risk of developing malignant tumors like hepatocellular carcinoma (HCC), which has been reported even at a young age in the Fontan population [8–10].

The development of FALD is subclinical with slightly abnormal to normal hepatic serological markers and does not reflect the often-severe hepatic structural changes that may appear within a few years after Fontan completion [11–13]. The mechanisms and development of this specific type of cardiac cirrhosis is still not fully understood. Currently, liver biopsy is considered the gold standard to evaluate liver fibrosis*/*cirrhosis of other etiologies. However, in Fontan patients the distribution of fibrosis*/*cirrhosis in FALD is often heterogeneous, which increases the risk of non-representative histological samples [14, 15]. Furthermore, a biopsy may be hazardous due to the risk for hemorrhage and general anesthesia for the procedure is often required in the pediatric age group [14, 16]. Imaging is suggested as a non-invasive alternative for surveillance of FALD, but its role, which modality to use, and at which interval are under debate [3].

Ultrasonography (US) is frequently used, although this technique is operator dependent and may give a suboptimal overview of the liver. Contrast enhanced CT and MRI are cross-sectional modalities where MRI has the advantage of providing tissue characterization further enhanced by using hepatobiliary contrast agents [17]. However, imaging findings are often difficult to interpret in this specific congestive hepatopathy. Advanced imaging techniques as ultrasound and magnetic resonance elastography as well as T1 mapping and diffusion-weighted imaging (DWI) have been suggested as tools for evaluation of the liver parenchyma and stiffness, and as non-invasive alternatives for liver biopsy [18–24]. However, these techniques are still under development and needs to be validated for FALD and pediatrics.

In this study, we aimed to register and categorize the spectrum of hepatic imaging findings on MRI in a defined national cohort of adolescents with Fontan circulation. We also compared the assessment of hepatic nodules on US versus MRI and performed a longitudinal observation of the nodules on MRI.

## Materials and methods

This single-center, cross-sectional study, with a prospective inclusion design, was approved by the Regional committee for medical and health research ethics (Nr 2013/1331), and registered in ClinicalTrials.gov NCT02378857. Written informed consent was obtained from all the adolescents and their caregivers.

We identified 67 patients with Fontan-type palliation of univentricular heart defects in Norway born between 1997 and 2002.

The study enrollment was performed during a 4–5-day hospital admission for routine diagnostic work-up at the pediatric cardiology department before transition to adult cardiology service. During day two, abdominal ultrasound and serological sampling was performed, and during day three, abdominal MRI was performed. Clinical data were collected from patient charts and biochemical serological analysis of liver function was registered.

### Ultrasonography

US was performed using a LogiqE9 (GE Healthcare, Chicago Illinois, US). All patients were instructed to fast for at least 2 h prior to the examination. The examination was performed with the patient in a supine position, using a 1–6 MHz probe curvilinear probe and a 2.5–8 MHz linear probe, by three dedicated pediatric radiologists (KST, KJT, CdL). The exam was performed as part of a larger US protocol where certain morphology criteria such as hepatic size, echogenicity, surface irregularity, the presence of nodules including their diameter were registered for this study (Table 1).

**Table 1:** Imaging criteria on magnetic resonance imaging (MRI) and ultrasonography (US)

Criteria	US	MRI
Irregular liver surface	Yes/no	Yes/no
Heterogeneous echo*/*signal structure	Yes/no	Yes/no
Hyperechogenic foci*/*nodules < 1 cm	Yes/no	Yes/no
Nodules ≥ 1 cm	Number	Number
Hepatomegaly		cm
Splenomegaly		cm
Ascites		Yes/no
Portosystemic collaterals		Yes/no

### MRI protocol

MRI was performed in all patients on the same 1.5 T Magnetom Aera (Siemens Healthcare, Erlangen, Germany) unit after a minimum of 3 h of fasting. A standardized protocol was set covering the liver and spleen combined with a cardiac MRI for a total work-up. A coronal T2 half-Fourier single shot turbo spin-echo (HASTE) and a single shot spin-echo-based echoplanar imaging sequence with diffusion-weighted imaging (DWI) sequences (*b* values 50-400-800) was performed pre-contrast. An axial T1-weighted 3D volumetric interpolated breath-hold (VIBE) examination Dixon was performed pre-, and post-contrast after administration of Gadoterate meglumine. A time-resolved angiography with interleaved stochastic trajectories over the thorax (which included the upper abdomen and the liver) was performed immediately prior to the 3D VIBE for the cardiac MRI protocol. Gadoterate meglumine was chosen as contrast agent since this is part of the standard protocol to evaluate liver lesions in our institution (Supplementary material).

The follow-up protocol was performed in the same 1.5 MR unit as above-mentioned except one exam in one patient performed at a local hospital, and included the above-mentioned abdominal sequences, using dynamic contrast enhancement with hepatobiliary contrast agent, Gadoxetic acid, including late hepatobiliary phase after 20 min (Supplementary material).

### MRI analysis

The images were interpreted by one radiologist (CdL) with 15 years’ experience and reviewed by another trained radiologist (KJT) with three years’ experience of pediatric abdominal MRI.

The assessment focused on imaging findings for hepatic fibrosis*/*cirrhosis according to the criteria shown in Table 1 [25]. Hepatic length was measured in the midclavicular line (in the coronal plane) where a length > 14.5 cm was considered pathologically increased [26, 27]. Splenic length was measured as the largest coronal diameter, where the craniocaudal length over 13 cm would indicate splenomegaly [28]. Ascites and portosystemic collaterals were registered, and rated as present or not.

The presence and the number of hepatic nodules were registered and the diameter was measured on the sequence they were best visualized on. Signal intensity compared to surrounding liver tissue on T1- and, T2-weighted images with and without fat suppression as well as on DWI, was recorded. DWI apparent diffusion coefficient (ADC) values were measured in the nodules by placing a region of interest as large as possible within the nodule, and one ≥ 1 cm in diameter, in the surrounding parenchyma. Contrast enhancement pattern was evaluated in arterial phase on the time-resolved angiography and in portal venous- and at delayed phase after 5 min.

All patients with hepatic nodules ≥ 1 cm in diameter on any sequence, were referred to an MRI examination within 1 month including dynamic hepatobiliary contrast administration for further characterization. Benign imaging features of nodules > 1 cm were defined as the absence of early ‘wash-out’ on portal venous phase as well as absence of ‘wash-out’ on delayed hepatobiliary phase and high signal intensity on T1-weighted imaging.

If imaging findings were benign and stable in size and MRI signal characteristics, since the first exam, these patients were further monitored with a repeat MRI again after 3 months and then at regular intervals, with MRI and US alternating every 6 months. The follow-up scheme was chosen to accord with published follow-up recommendations for FALD at the time of the study and adjusted in consensus to our hospital guidelines for follow-up of HCC suspicious liver nodules in chronic liver disease [29–33].

### Hepatic biomarkers

Blood samples were drawn in the morning of day two of the hospital admission. Albumin, international normalized ratio (INR), total bilirubin, bile acids, aspartate transaminase, alanine aminotransferase, alkaline phosphatase, gamma-glutamyl transferase, lactate dehydrogenase and alpha fetoprotein were analyzed and chosen as recommend in consensus statements [3, 33]

### Statistical analysis

Data were presented as mean ± standard deviation or median with interquartile range, as appropriate. We performed the comparisons of continuous data by the unpaired Student’s *t*-test or Mann–Whitney *U* test as appropriate. Categorical variables were compared by the *χ*^2^ test or Fishers’ exact test. Two-tailed *p*-values ≤ 0.05 were considered statistically significant. Statistical analyses were performed with SPSS version 26 (IBM, Armonk, NY, USA).

## Results

### Patient characteristics

Of the 67 identified patients, thirteen patients were excluded due to; death *n* = 4, a heart transplant performed or on waiting list *n* = 2, move abroad *n* = 2 or recent cardiac work-up *n* = 2 and severe neurological impairments *n* = 3. Finally, 54 adolescents (15–17 years) with Fontan circulation were invited to participate in the study from March 2015 to December 2018. Of these, eight finally declined inclusion and the remaining 46 patients with median age of 16.5 years (range 15.4–17.9 years) where included (18 girls). Eleven patients could not complete the MRI examination either due to claustrophobia (*n* = 3) or having non-compatible pacemakers and*/*or leads (*n* = 8). In total, 46 patients completed the US and 35 patients completed the MRI examination. None of the patients had any reported hepatic disease prior to examination. The patients had a variety of univentricular defects of whom 24 patients had a morphologically single left and 19 had a morphologically single right ventricle. Three patients had anatomically two ventricles functioning as a single ventricle. One patient had left isomerism with polysplenia, and one had situs inversus abdominis. Patient characteristics are displayed in Table 2.

**Table 2:** Patient characteristics and hepatic serological markers

Characteristics	Mean value ± SD
Clinical/surgical *n* = 46	
Age at MRI/US (years)	16.7 ± 0.6 (15.4–17.9)
Gender male *n* (%)	28 (61%)
Weight (kg)	58.2 ± 11.4
BSA (m^2^)	1.6 ± 0.2
Age at Fontan operation (years)	2.6 ± 2.0
Interval MRI/US to Fontan operation (days)	5021 ± 1076
Heart defect	
Hypoplastic left heart	11
Tricuspid atresia	9
Pulmonary atresia	7
Double inlet left ventricle	6
Other heart defect	13
Morphological left/right*/*common ventricle	24/19/3

Hepatic serological markers *n* = 45	Mean ± SD	Median
Metabolic		
Alanine aminotransferase (U/L)	31.7 ± 14	28
Aspartate transaminase (U/L)	29.4 ± 7.7	28
Gamma-glutamyl transferase (U/L)	72.2 ± 37.7	63
Bilirubin (µmol/L)	13.7 ± 9.2	13
Bile acid (µmol/L)	12.2 ± 19.1	6
Lactate dehydrogenase (U/L)	181.8 ± 30.2	182
Alkaline phosphatase (U/L)	134.9 ± 51	115
Synthetic		
Albumin (g/L)	45.8 ± 5.1	46
International normalized ratio (except warfarin users)	1.2 ± 0.1	1.1
Oncologic		
Alpha Fetoprotein (10E3 U/L)	2.7 ± 1.5	2

*MRI* magnetic resonance imaging, *SD* standard deviation, *US* ultrasonography

Three patients were concluded to have protein-losing enteropathy as part of a failing Fontan circulation, based on a combination clinical symptoms and biochemical markers (hypoalbuminemia, hypogammaglobulinemia, edema, and ascites).

### Ultrasonography findings

Of the 46 patients 26% (*n* = 12) had a normal hepatic structure without focal lesions, while 74% had various abnormal of findings.

**Fig. 1:**
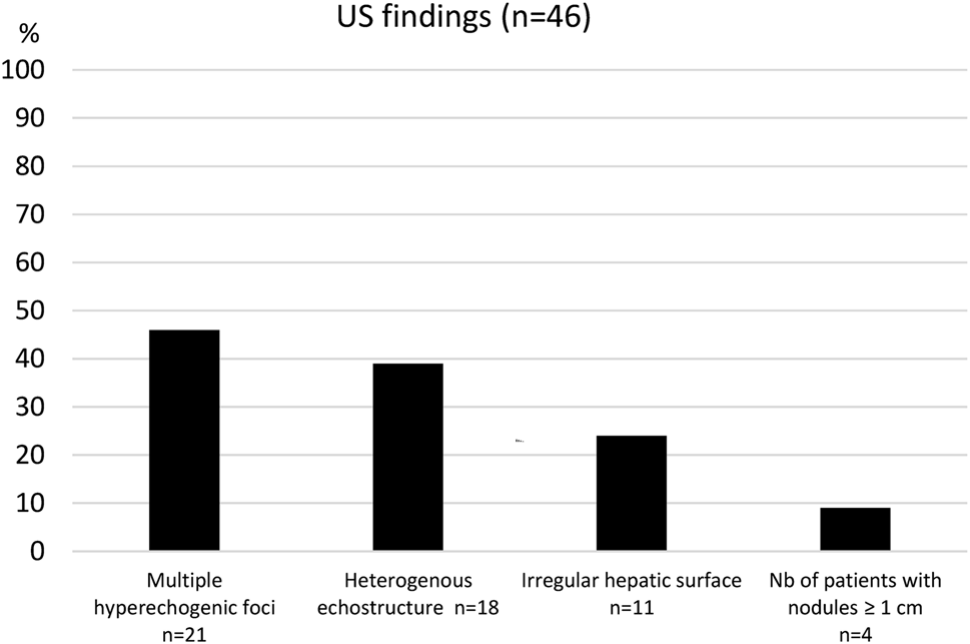
Frequency of different ultrasonography (US) findings

**Fig. 2:**
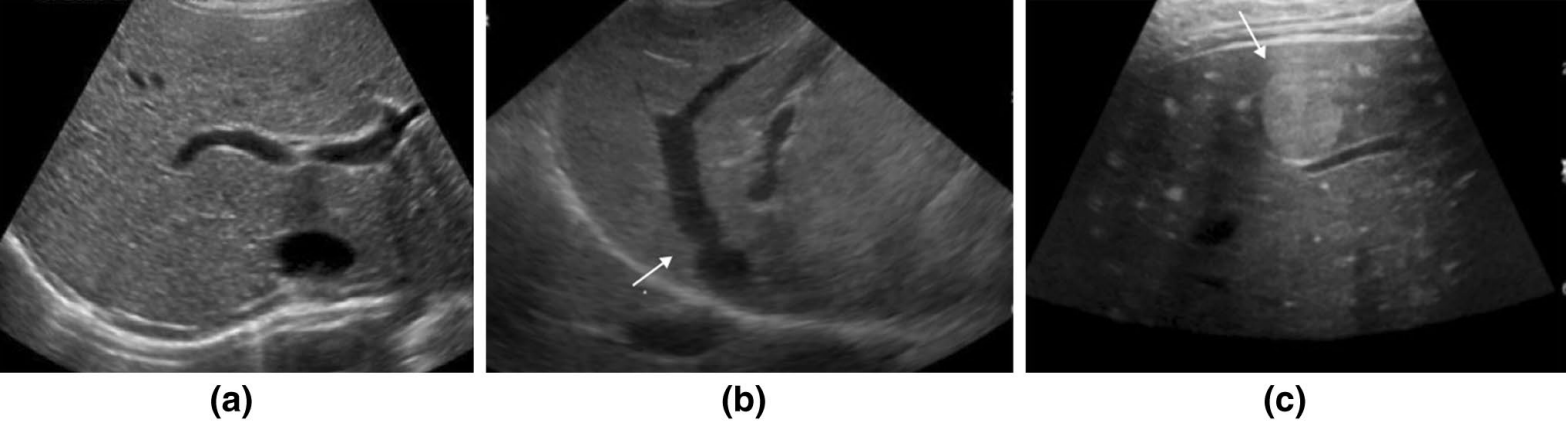
Adolescents 15–17 years of age with different findings on US **a**–**c**. **a** Normal echogenicity of the liver, **b** hepatic congestion with a dilated liver vein (arrow), **c** Multiple hyperechogenic small and one larger nodular lesion (arrow)

Coarse heterogeneous echo structure was found in 39% (*n* = 18), and an irregular liver surface in 24% (*n* = 11). Nodules of different size were frequent, and 46% (*n* = 21) had multiple small 2–3 mm hyperechogenic foci. In three patients, four hepatic nodules ≥ 1 cm in diameter were seen. (Figs. 1 and 2).

### Magnetic resonance imaging findings

**Fig. 3:**
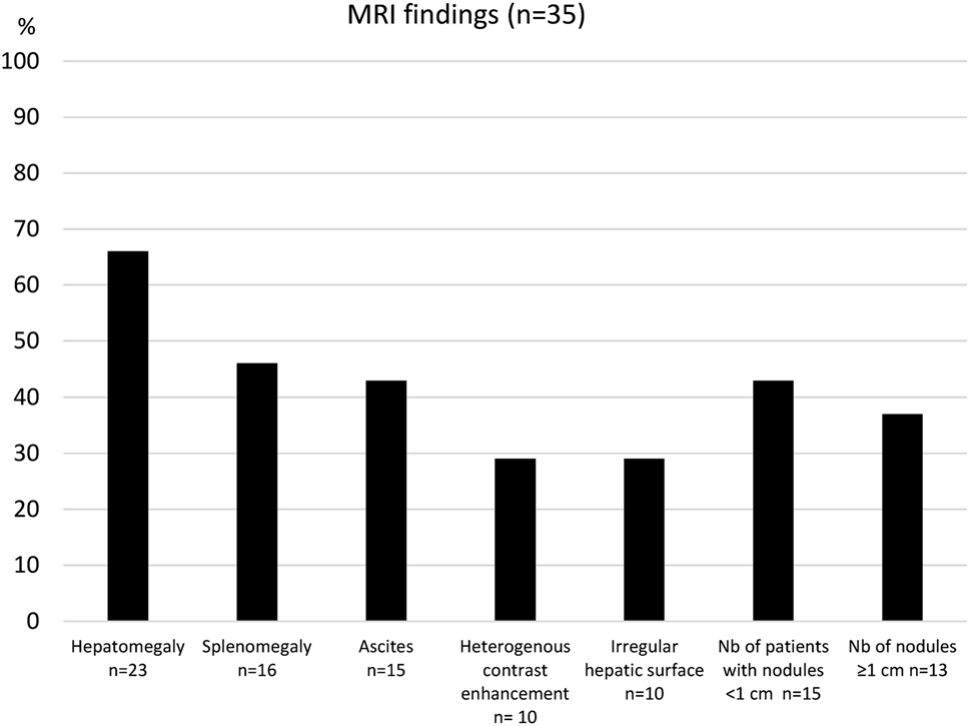
Frequency of different magnetic resonance imaging (MRI) findings

**Fig. 4:**
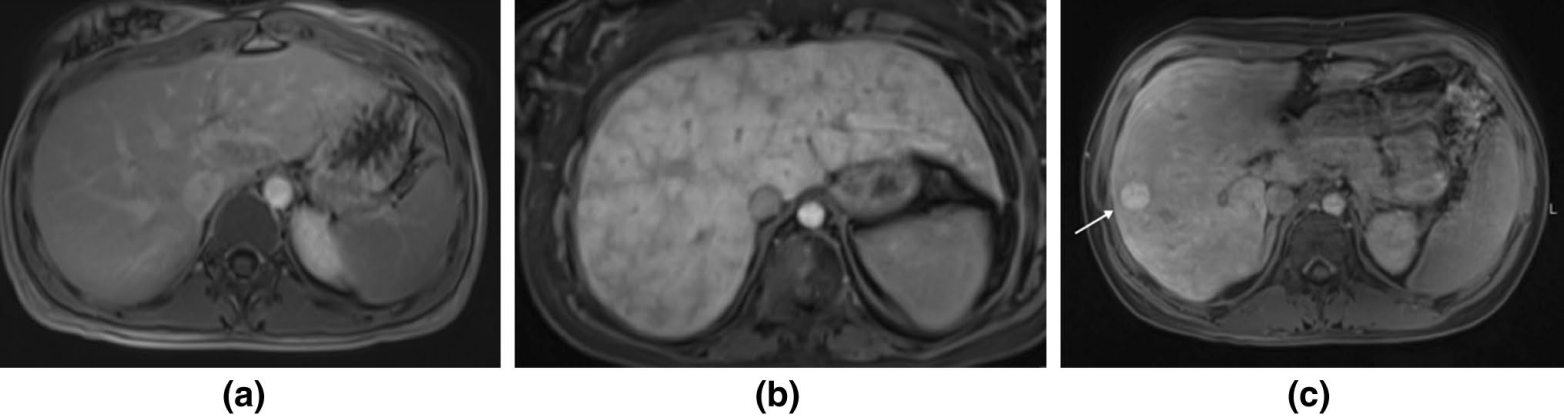
MR T1 gradient echo sequence post-contrast in portal venous phase **a**–**c**. **a** Normal signal of liver parenchyma, **b** hepatic congestion and typical perisinusoidal contrast enhancement. **c** irregular hepatic surface and atrophy of left liver lobe as in cirrhosis with enhancing nodular lesions, one larger in the right liver lobe (arrow)

**Fig. 5:**
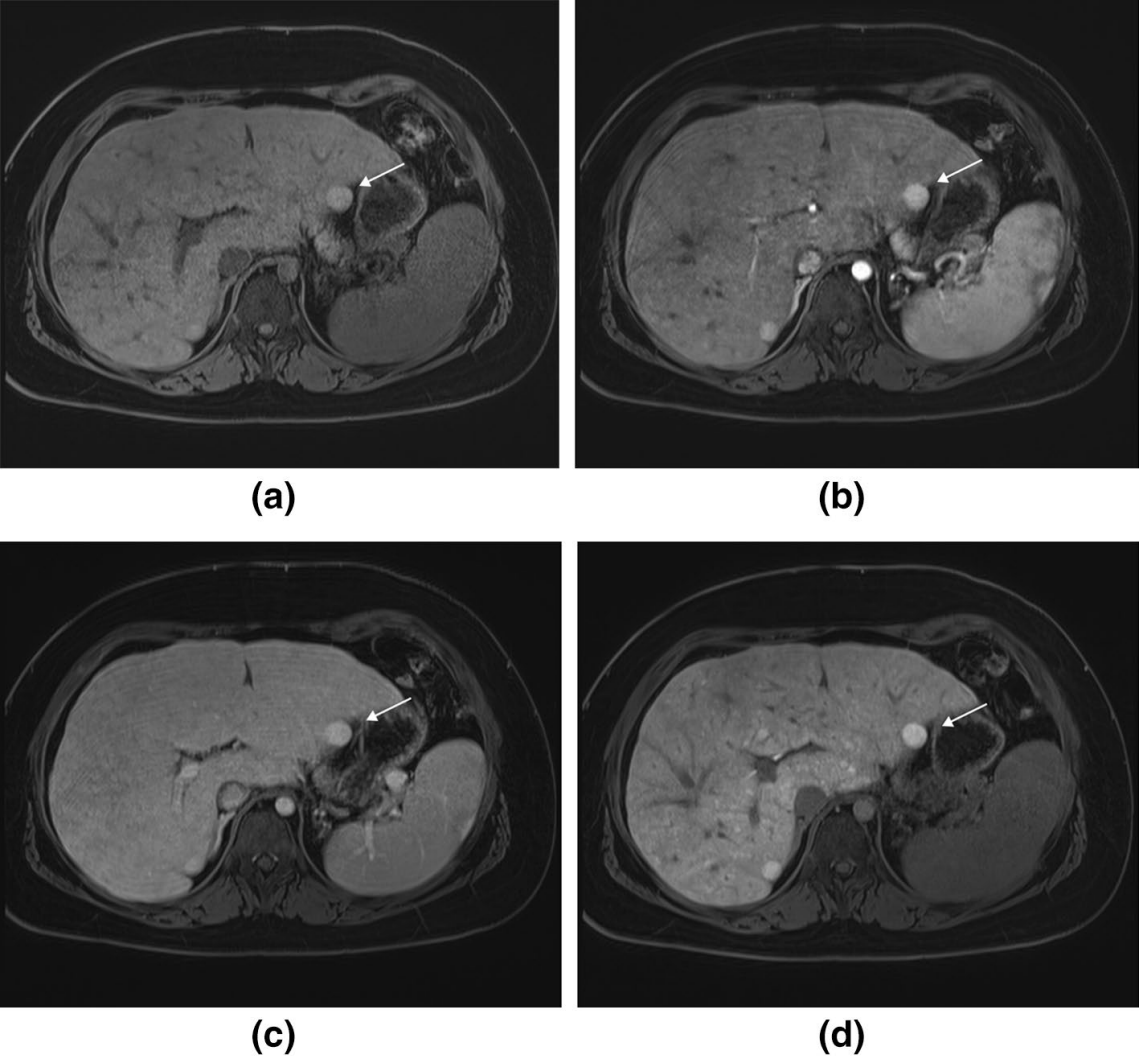
MRI of the liver of a 15-year-old girl with Fontan circulation and a morphologically left single ventricle. Axial T1-weighted gradient echo sequence with fat suppression reveals an enlarged liver with congestive pattern and irregular hepatic surface with a nodule 13 mm in diameter, in the left liver lobe (thin arrows) with a hyper/isointense signal compared to liver parenchyma. Splenomegaly in **a**. Contrast enhancement with hepatobiliary contrast agent, gadoxetic acid; the nodule with hyperintense signal in arterial phase in **b** and in portal venous phase **c**. Strong enhancement of the nodule in hepatobiliary phase and additional appearance of multiple other small enhancing nodules, in **d**. Gradient echo images in and out of phase did not reveal fat in the nodule (images not shown). The lesion was interpreted as a focal nodular hyperplasia-like lesion and remained stable in size and signal during follow-up of 3.5 years

The length of the liver was, median 15.1 cm (11.5–19.4) where 21/35 had a length > 14.5 cm. Sixteen out of 35 had spleen length > 13 cm median 12.8 (8–18.8 cm). Ten patients had an irregular hepatic surface. Heterogeneous contrast enhancement in the portal venous phase with a peripheral reticular enhancement pattern was noted in ten patients. Seven patients had an irregular hepatic surface and irregular parenchymal T1 and T2-weighted signal (Figs. 3, 4, 5).

None of the subjects presented with portosystemic collaterals. Ascites was registered in fifteen patients of whom thirteen only had a minimal amount in the pelvis. Two of the subjects had severe amount of ascites around the liver, spleen, and in the pelvis.

Fifteen patients had multiple small nodules < 1 cm in diameter with high signal on T2-weighted images. Seven patients had together thirteen nodules ≥ 1 cm in size with a median diameter of 13 mm (10–23 mm). Three patients had both small and larger nodules. The nodules showed variable signal characteristics on different MR sequences between different nodules within the same liver (Table 3). On DWI, ADC values in nodules had a mean value 1111 ± 124 10^–6^ mm^2^/s (median 1091, range 910–1291 10^–6^ mm^2^/s). The surrounding liver parenchyma had a heterogeneous diffusion pattern with mean ADC values of 1051 ± 55 10^–6^ mm^2^/s (median 1035, range 853–1314 10^–6^ mm^2^/s).

**Table 3:** MR signal and contrast enhancement characteristics of nodules ≥ 1 cm

MR sequence and signal intensity compared to liver	First exam*/*follow-up *n* (%)	Last follow-up *n* (%)
T2-weighted sequence	*n* = 13	*n* = 11
Hypointense	4 (31)	3 (27)
Isointense	9 (69)	8 (73)
Hyperintense	0	0

T1-weighted sequence	*n* = 13	*n* = 9
Hypointense	1 (8)	0
Isointense	7 (54)	6 (67)
Hyperintense	5 (38)	3 (33)

Diffusion-weighted imaging	*n* = 11	*n* = 11
Restricted	0	0
Same as the liver	3 (27)	10 (91)
Increased	8 (73)	1 (9)

Contrast enhancement pattern	*n* = 13	*n* = 11
Arterial	12 (92)	11 (100
Portal venous isointense	11 (85)	11 (100)
Portal venous wash-out	2 (15)	0
Delayed phase hyperintense	1 (10)	0
Delayed phase isointense	7 (70)	11(100)
Delayed phase hypointense	2 (20)	0
Hepatobiliary phase (20 min) Hyperintense	9(100) *n* = 9	5 (83%) *n* = 6

The number of nodules examined at first exam*/*follow-up was (*n* = 13) and at the last follow-up (*n* = 11), since two nodules were lost for follow-up. Diffusion-weighted imaging was possible to interpret in *n* = 11. Contrast enhancement was evaluated in *n* = 13 at first exam*/*follow-up (only *n* = 10 in delayed phase) and in *n* = 11 at last follow-up. Hepatobiliary contrast enhancement was evaluated in *n* = 9 at first follow-up and in *n* = 6 at the final exam

**Fig. 6:**
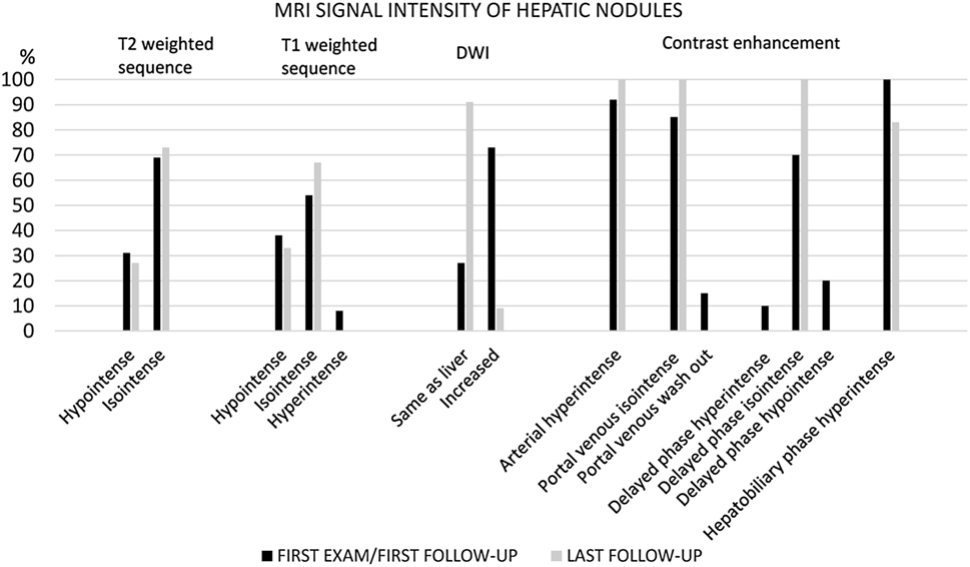
Magnetic resonance imaging (MRI) signal characteristics of 13 nodules ≥ 1 cm in diameter

**Fig. 7:**
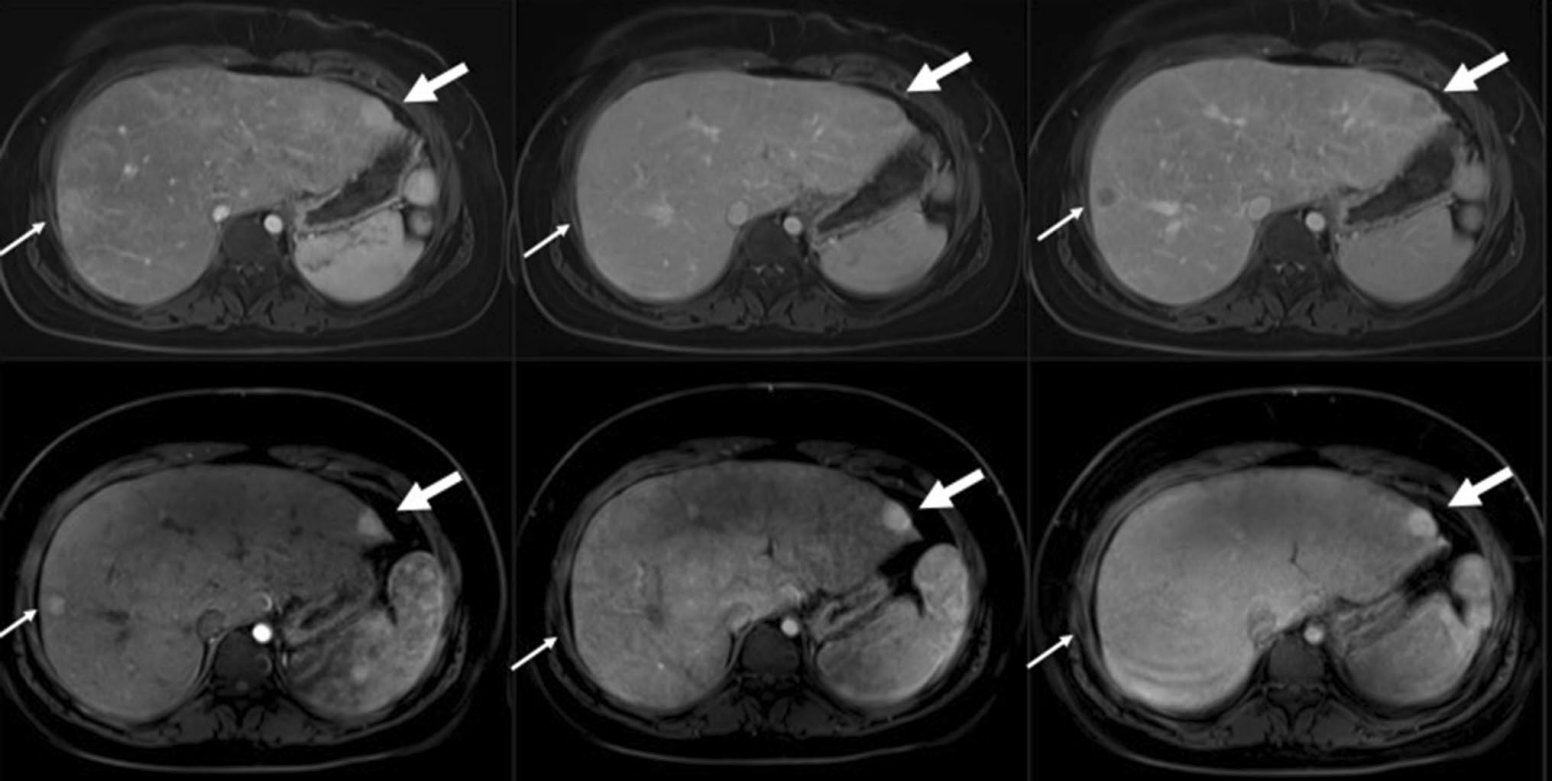
MRI of the liver of a 15-year-old girl with Fontan circulation and a morphologically left single ventricle. This is the same patient as in Fig. 4 showing contrast enhancement in two other nodules, one in the lateral part of the right liver lobe (thin arrows) and one in the left liver lobe (thick arrows). The upper row represents the initial examination with gadoterate meglumine and the lower row the first follow-up examination with gadoxetic acid. Images from left to right are in late arterial*/*arterial phase, portal venous phase and delayed portal venous phase. The initial examination reveals arterial enhancement of the two nodules with an isointense signal in the portal venous phase and a clear ‘wash-out’ in the delayed portal venous phase. In the follow-up examination, the portal venous and delayed phases display a homogeneous contrast enhancement of the nodules

Contrast enhancement was evaluated in most patients and arterial hyperenhancement of the nodules was an overall finding (Table 3 and Fig. 6). In the portal venous phase most nodules ≥ 1 cm were isointense to liver, except two presenting with ‘wash-out’ pattern, remaining hypointense in the delayed phase (Table 3, Figs. 6, 7). This patient with two nodules was referred to follow-up with MRI including hepatobiliary contrast administration for further characterization for suspicious of carcinoma (Fig. 7), (see section below).

### Follow-up findings in nodules

Of the seven patients with together 13 nodules ≥ 1 cm, follow-up data were available for five of them. The two patients with unavailable data presented each with a single nodule with MR signal and enhancement pattern interpreted as benign on the initial scan.

The remaining five patients with in total eleven nodules, were monitored repeatedly with MRI, with the last exam performed before transition to adult congenital heart disease care after a median 24.4 months (7–42 months) from first examination. The number of follow-up MR examinations varied from 2 to 4 exams before they were left to adult cardiology service and the number depended on their age at time of inclusion. At the follow-up investigation, no nodules had clear imaging findings suspicious of hepatocellular carcinoma*/*adenoma. Changes in signal characteristics at the last follow-up are displayed in Table 3 and Fig. 6. Some nodules increased a few mm in diameter (*n* = 5), decreased (*n* = 2) or remained unchanged in size (*n* = 4) during follow-up of up to 3.5 years.

### Hepatic biomarkers

Plasma biomarkers were available 45 out of 46 patients (Table 2). Only 6 patients had aspartate transferase above 50 U/L (range 19–66) or alanine aminotransferase above 50 U*/*L (range 10–71). The international normalized ratio was normal (up to 1.3) in all patients not receiving warfarin. Three patients had protein-losing enteropathy where 2 had markedly reduced albumin levels (both 23 g/L),and one patient had slightly reduced albumin 36 g/l. The level of alpha fetoprotein was within normal range < 14 10E3 U/L for all patients. Gamma-glutamyl transferase was slightly elevated with 19 patients having > 60 U/L (range 26–194).

Gamma-glutamyl transferase was the only marker with significant difference between patients with a morphological single right vs left ventricle 87 ± 43 vs 54 ± 23 *p* = 0.004.

Concerning the correlation between the presence of nodules and the different serological markers significant weak correlations were found with alkaline phosphatase with *R* = − 0.37, *p* = 0.01 and bilirubin *R* = − 0.30, *p* = 0.03. There were neither significant correlation to other serological markers nor to imaging findings of fibrosis*/*cirrhosis as presented in Table 1, all with *R* < 0.3, *p* > 0.05 (results not presented).

## Discussion

This is a single-center, cross-sectional study, prospectively including a population-based sample of adolescent patients with Fontan circulation. We describe the range of hepatic imaging findings in US and MRI, including findings on a longitudinal follow-up of a median of 24.4 months in a subgroup. This gives important insight into the heterogeneous hepatic appearances in this cohort of 15–17-year-old patients on the transition to adulthood.

Our main results were; (a) imaging findings of hepatomegaly with edema, fibrosis and cirrhosis with nodules are frequent in these young adolescents and of various degree of severity. (b) US had a low sensitivity for identifying hepatic nodules compared to MRI. (c) Nodules have variable MRI signal characteristics some with changing features over time. (d) Hepatic nodules ≥ 1 cm had benign contrast enhancement pattern including hepatobiliary agent except in one patient where two nodules presented with ‘wash-out’ in portal venous phase. However, no imaging findings or serologic markers of malignancy were found during up to 3.5 years follow-up (Fig. 7).

### Imaging of Fontan-associated liver disease and serological markers

FALD expresses both sinusoidal and portal fibrosis caused by hepatic congestion and ischemia and this unique derangement of hepatic architecture leads to distinct radiological features [21, 32]. Both MRI and US display hepatomegaly, nodular surface and heterogeneous echogenicity and MRI signal, while the contrast enhancement pattern is often heterogeneous reflecting the hepatic congestion. Findings of hepatic nodules are also an important feature, mostly represented by regenerative nodules and focal nodular hyperplasia-like nodules [34, 35]. Early onset of hepatic fibrosis/cirrhosis and the risk of developing HCC already at young age is a concern [9, 10]. Ultimately, advanced end stage FALD with a malignancy may prohibit a heart transplantation which is the only curative treatment for univentricular heart defects, or even demand for a combined transplant of the heart and liver [31].

The prevalence of imaging findings of hepatic disease in our study cohort is high. Seventy-four percent of the patients had either coarse echo structure, surface nodularity, multiple hyperechogenic nodules, or a combination of the three on US and corresponding findings on MRI (Figs. 1 and 3). The contrast enhancement pattern is typically/often seen in FALD, especially in the periphery of the liver with a distinct reticular, patchy pattern of enhancement during the late arterial and portal venous phases which equilibrates with background liver during the delayed phase. This phenomenon is presumably due to delayed wash-in of contrast material into the congested liver [21, 35, 36]. These findings of FALD are in line with several previous studies and reports, describing the same features but in contrary to our material, their study groups represent a large age-range, mainly adults and often with small sample sizes [21, 25, 37, 38]. It has been shown that patients have variable development and severity of FALD at different ages and following time since the Fontan operation with individual variability [39]. Our homogeneous study group with a relatively large number of teenagers exclusively and within a narrow age span represent a national cohort from a country with a uniform healthcare system and follow-up. Under these circumstances one could have expected an in general less advanced and more uniform degree of FALD. However, their imaging findings were markedly heterogenous in degree and in many cases presenting with signs of advanced liver disease. The lack of markedly elevated hepatic serologic markers in our cohort is consistent with previous findings in patients with Fontan circulation, where normal levels of conventional biomarkers were observed, despite severe structural liver changes [3, 7, 37, 38].

### Imaging characteristics of liver nodules

As described by other authors the most common finding were the small 2–3 mm hyperechogenic nodules dispersed in the parenchyma with high MRI signal on T2-weighted images and arterial enhancement [21, 38, 40]. They are thought to be the result of local liver injury and altered blood-flow with arterialization [35, 41]. Arterial enhancement was the most common MRI finding of the nodules. Over 50% of the nodules had isointense signal to liver on T2 and T1 which is earlier described as a typical MRI pattern of hepatic nodules encountered in congestive hepatopathy [36].

However, two nodules in two different patients had high signal on T1 and low on T2-weighted sequence. Their contrast enhancement was predominantly arterial, and where isointense to liver in portal venous phase. These are atypical features for benign nodules, and the signal pattern, could be explained by an abnormally increased background signal pre-contrast and enhancement post-contrast due to congestion [34]. This has also been found by studies and experiences from other authors, suggesting that biopsy should be performed on atypical nodular lesions. [25, 34, 38].

On DWI, all of the nodules had similar or increased diffusion as compared to the surrounding liver tissue, and not restricted which is usually associated with highly cellular tumors. A study by Wolff et al. studied DWI in the whole parenchyma of 59 Fontan patients, both children and adults, found lower ADC values in the liver than reported in normal subjects [22]. Others report of adult cases with nodules having similar non-restricted diffusion pattern compared to the parenchyma, as in our patients [36, 42]. However, our cohort has few nodules with a variable range of measurements and ADC values in the nodules by large overlapped with extra-nodular values hence our findings support previous research suggesting that DWI*/*ADC is insufficient on its own to distinguish malign from benign lesions, and especially not in congestive hepatopathy [21].

In a retrospective multicenter study by Egbe et al. the prevalence of HCC in the Fontan population was found to be 1.3%, and the youngest patient was only twelve years old [8]. Similar findings in a large prospective multicenter study of 152 patients revealed only two patients with biopsy proven HCC [38]. In retrospect and in light of recent reports we could have considered to biopsy the two nodules with ‘wash-out’ contrast enhancement pattern despite normal levels of alpha fetoprotein. At the time of the study, the risk of complications to this intervention was considered as a too high and in consensus with the clinical team, we chose to perform close follow-up with MR examinations. Findings revealed the nodules with stable size and MRI signal without portal venous ‘wash-out’ and with contrast enhancement on hepatobiliary phase, on four repeated MRI exams under 3.5 years follow-up (Fig. 7). However, it is not clear if HCC develops from hepatic nodules or if they appear independent of them [42]. Mazzarelli et al. revealed that HCC was registered in three patients without association to the degree of hepatic cirrhosis [43] which was also confirmed in a meta-analysis by Rodriguez et al. [44]. In our study we found that larger nodules were present even without imaging criteria for cirrhosis and not uncommonly in a liver with otherwise normal MR signal and US echogenicity. On the other hand, focal liver lesions in Fontan may reside on a continuum with carcinomas and arise from adenomatous lesions.

In summary, the different imaging characteristics of these nodules might reflect the broad variation of hepatic pathology and the different stages of fibrosis*/*cirrhosis in this patient group, although the number of patients and nodules are probably too small to make a clear conclusion.

### Sensitivity of imaging techniques for detection of hepatic nodular lesions

Abdominal US is a widely available and cheap examination which poses no risk of radiation, and should be a first choice of assessing the liver in this young population. Ultrasound may detect signs of fibrosis*/*cirrhosis like changes in liver size, splenomegaly, nodular liver surface and coarse heterogenous echogenicity. However, in our study US only visualized 31% of the nodules seen on MRI. The nodules are often isoechoic to surrounding parenchyma with hepatomegaly and coarse hepatic echo structure which makes them challenging to visualize on US even when scanning with a linear probe [21, 38]. In the study by Horvat et al. MR could detect six times as many lesions as US [45]. However, despite US being far inferior to MRI in detecting liver nodules, in the study by Tellez et al., US did not miss any case of hepatocellular carcinoma. Hence, more studies are warranted before US could be ruled out as a screening tool [38].

At the time of our study we aimed to monitor our patients with larger nodules to investigate and monitor for a malignancy, not to miss out on possible precursors of HCC, according to current recommendations, the latest from the American Heart Association scientific statement [3, 29, 30]. However, based on our findings as well as others experiences, FALD is variable and independently associated with Fontan duration and it is difficult to recommend monitoring intervals to detect early malignancy [21, 38, 46]. On the other hand, to study adolescents at 15–18 years of age mean to describe the disease state at a crucial age that marks transition from pediatric to adult care. The importance of involving hepatology expertise in the team caring for young adults with Fontan circulation is strongly underlined by our data*.* Monitoring the development of liver fibrosis*/*cirrhosis even without malignancy, may be of importance also as a part of the assessment for timing of heart transplantation.

## Limitations

The study was performed as a national cohort study, but the sample size remains small with the possible accompanying statistical limitations.

Due to the unreliable histologic sampling of the heterogeneous FALD parenchyma and risk of complications we did not perform liver biopsy as a part of the study protocol. The nodules ≥ 1 cm in size, all had a total work-up and imaging features favoring a benign lesion. They were therefore not histologically verified.

The total time of surveillance of patients with larger nodules with MR was limited to between 7 months and 3.5 years. This was mainly due to the transition of the patients to adult care of congenital heart disease where unfortunately a strict follow-up regime was not established at the time of the study.

## Conclusion

In a national cohort of young adolescents with Fontan palliation, hepatic changes including nodules presented on MRI and US, were highly prevalent. At the time of transition to adult congenital heart disease care, no signs of malignancy were found. US had a low sensitivity to detect nodules even ≥ 1 cm which is an important indication to perform an MRI. The imaging findings of advanced liver disease found in our young patients, confirms that long-term surveillance of Fontan palliated pediatric patients is mandatory. To monitor the development of liver fibrosis cirrhosis even without malignancy may be of importance in the assessment of timing for heart transplantation. In this regard, a multidisciplinary approach is important involving not only cardiologists and radiologists but also hepatologists and eventually other specialized physicians. The outline of imaging surveillance and examination intervals needs to be further determined in longitudinal studies.

## Supplementary Information

Below is the link to the electronic supplementary material.

Electronic supplementary material 1 (DOCX 14 kb)
