# Supplementary material for: Spectrum of Fontan-associated liver disease assessed by MRI and US in young adolescents
Source: Abdom Radiol (NY). 2021 Mar 10;46(7):3205–16. doi: 10.1007/s00261-021-02994-0 (PMC8215034; doi:10.1007/s00261-021-02994-0)
Supplement: Supplementary file 1 — Electronic supplementary material 1 (DOCX 14 kb) [file 261_2021_2994_MOESM1_ESM.docx]

## Supplementary material

*MR protocol initial scan*

A coronal breath hold (BH) T2 half-Fourier single shot turbo spin echo (HASTE) with the following parameters: repetition time (TR) 1200 ms; echo time (TE) 82 ms; flip angle 180°; field of view (FOV) 350 mm; generalized autocalibrating partially parallel acquisition (GRAPPA) acceleration of 2 with 24 reference lines with a voxel size of 1.4 x 1.4 x 5 mm; acquisition time (TA) 41 s.

An axial T1 3D Volumetric interpolated breath-hold examination (VIBE) Dixon, pre/post- contrast with the following parameters: TR 6.74 ms; TE1 2.39 ms and TE2 4.77 ms; flip angle 15°; FOV 360 mm; controlled aliasing in parallel imaging results in higher acceleration (CAIPIRINHA) of 3 with 24 reference lines with a voxel size of 1.2 x 1.2 x 3 mm; TA 16 s.

An axial single shot spin-echo based echoplanar imaging sequence with three diffusion weighted imaging (DWI) series, b50-b400-b800 s/mm², with the following parameters: TR 6300 ms; TE 47 ms; FOV 368 mm; GRAPPA acceleration of 2 with 36 reference lines with a voxel size of 2 x 2 x 5 mm; TA 300 s.

A coronal time-resolved angiography with interleaved stochastic trajectories (TWIST) after administration of 0,2 ml/kg Gadoterate meglumine injection rate 1.3 ml/s “over 2 min” with the following parameters: TR 2.55 ms; TE 0.91 ms; flip angle 25°; FOV 380 mm; GRAPPA acceleration of 2 with 24 reference lines with a voxel size of 1.3 x 1.3 x 1.5 mm. Immediately after TWIST, a new dose of 0.2 ml/kg Gadoterate meglumine was injected, where the T1 VIBE sequences post contrast in arterial, portal venous and late portal venous phase was acquired.

*MR protocol follow-up scan*

A coronal breath hold (BH) T2 half-Fourier single shot turbo spin echo (HASTE) with the following parameters: repetition time (TR) 1200 ms; echo time (TE) 82 ms; flip angle 180°; field of view (FOV) 350 mm; generalized autocalibrating partially parallel acquisition (GRAPPA) acceleration of 2 with 24 reference lines with a voxel size of 1.4 x 1.4 x 5 mm; acquisition time (TA) 41 s.

An axial T1 3D Volumetric interpolated breath hold examination (VIBE) Dixon, pre/post- contrast with the following parameters: TR 6.74 ms; TE1 2.39 ms and TE2 4.77 ms; flip angle 15°; FOV 360 mm; controlled aliasing in parallel imaging results in higher acceleration (CAIPIRINHA) of 3 with 24 reference lines with a voxel size of 1,2 x 1,2 x 3 mm; TA 16 s. Gadoxetic acid was used as contrast agent, 0.1 ml/kg and injection rate of 1 ml/s, where the T1 VIBE sequences post contrast in arterial, portal venous and hepatobiliary phase was acquired

An axial spin-echo based echoplanar imaging sequence with three diffusion weighted imaging (DWI) series, b50-b400-b800 s/mm², with the following parameters: TR 7400 ms; TE 59 ms; FOV 368 mm; GRAPPA acceleration of 2 with 36 reference lines with a voxel size of 1 x 1 x 5 mm; TA 300 s.
